# Embryology

**Published:** 1878-09

**Authors:** W. H. Atkinson


### EMBRYOLOGY.

BY DR. W. H. ATKINSON.

*Read before the American Dental Convention.*

A complete understanding of embryolgy involves the comprehension of the processes of nutrition, whereby parent bodies become possessed of a plus quantity of well elaborated nutrient material to enable them to project buds, the shedding and coalescence of which constitute the semens from which, when these flow together under favorable circumstances, embryos result. The elaboration of the seminal mass and egg consists of an adequation of molecular tensions, or the record (storing up) of the atomic experiences in combination with, and separation of, the atoms in producing the molecules of which the food consists, from which the semen is derived as the resultant of a complicated series of digestory and secretory processes.

Naturalists have observed from the earliest times that "like begets like," and "unlike begets difference," wherever germs result from the admixture of semen and egg, or male and female semens derived from well-nourished bodies. That which we call inheritance is none other than the recorded psychical and molecular experiences of the parents preserved in the semens. The re-awaking of this "past molecular experience" of the parents, is the basis of development of the progeny, wherever it is free to express itself. The simplest form of parentage we are acquainted with consists in the flowing together of atoms, which conceals their identity, in the molecules they constitute as progeny, by the mergence of their atomic presence into molecular mass. From this starting point of parent and progeny, we gradually rise through various stages, complications and refinements in molecular changes, until the parent is able to produce a progeny without losing its own identity.

Molecular metamorphosis have many degrees of interblendings in the tension of bonds which constitute ripeness of the mass, in an equation of the satisfaction of the desire to unite, from the lowest and simplest pabulum to the highest and finest semen. Thc first is exampled in water; the last in the dual presentment of animal spirit in the gray matter of the cerebrum and cerebellum, as the copulative basis of thought and feeling, consciousness and physics, or mind and its medium.

The molecular mass with which we have to do in the study of embryology is derived from the food upon which the parent bodies subsist, by a process of digestive metamorphosis, which adapts it to the necessities of the tissues to be supplied with the elements of growth and maintenance. The materials of food, their preparation and the surrounding circumstances under which it is eaten and digested, are important items in the production of the pabulum upon which the tissues are fed or starved. That which has been called over-feeding may be starving, by reason of the interference with the blood-making and force-generating apparatus. Many mothers are starved during pregnancy both in body and soul, and many fœtuses are, on the other hand, starved by reason of misdirection of nutrient currents in the pabulum through bad habits of the mother. These derangements are attributable to a variety of incidents: want of quantity or quality of food supply, inharmonious associates during digestion and gestation, are examplesof physical and moral antecedents of imperfectly elaborated pabulum and illy digested progeny.

All the secretions are under the law of reflex nervous incitement, and therefore partaking of the nature of the source of the exciting impulse, thus *di*vinizing or *de*vinizing the product of the secretory apparatus all the way from saliva to semen, in testes, ovary and brain. These potentialities are stored in the secretions, and only await the opportunity to display themselves.

That the character of a secretion partakes of the nature of its exciting circumstance, is proved by the quality of the saliva being different in diverse states under which it is produced. Pleasant states of mind induce alkaline saliva; angry persons secrete acid and viscous saliva, or are unable to secrete from these glands at all. Sugars and starches demand alkaline saliva, while nitrogenous substances have next to no power to invoke other than a neutral saliva.

The presence of persons has an unconscious influence upon our secretions, and hence influence us on the plane of mere vegetative, involuntary, or "unconscious-consciousness," or "molecular-consciousness," thus modifying our physical and moral states before we are aware of it. Our natural likes and dislikes are indices of these states, and we should heed them if we would be healthy ourselves, or produce healthy and well developed progeny. The old saw, "Everybody loves a lover," is the testimony of the ages to this doctrine of "impartation" of our condition to those within our "sphere of influence." Mere *personal presence* often *heals*; and alas! it sometimes *hurts* almost irreparably, because of the occultness with which its malign force operates. Who has not witnessed sunshine and joy irradiate a company upon the entrance of one full to overflowing with life and hope; and who has not seen the shades of sadness and shrinking seize a company of happy souls, upon the advent into its sacred precincts of a well-known dreaded "Kill-Joy," who has determined within his unhappy self, "If I can not be thus joyous, neither shall you!" and until that dark spirit has been expelled, who has ever seen health and peace prevail as was its wont? How many of us are ready to acknowledge that our real life "is hidden in truth in the unseen realms" of our interior-posterior brain and glangionic system of nerves, more than it is in our exterior-anterior superior brain, where we but record and arrange the perceptions spontaneously given by the interior grey cells of the cerebellum and sentient neural system?

Whether ready to acknowledge it or not, we are as embryos and adults, much more at the mercy of surroundings and registered "past experiences" of ancestry than our egotism leads us to accept. Could all these past experiences be plainly set before our present perception, we would be in possession of the values of all the changes of integration and disintegration of the elements of mental and bodily machinery. But such has been the loose habit of observation in this direction, that we have actively retained in our surface memory the merest fraction of the experiences through which we have passed, and we are thus induced to claim for ourselves a degree of freedom and responsibility by no means justified in the history of our embryonal and adult experiences. Were we to assert that consciousness never loses any of its experiences, we might be answered by a denial of the truth of the assertion, and be required to justify its verity by a recapitulation of the entire series of our past experiences, which could only be done by a reference to the multitude of items, in the experiences involved in any history, or consume as much time in the statement as was formerly occupied in the enactment of this history.

Of the three distinct sets of currents which constitute adult somatic functions, two only are well pronounced in intra-uterine development. These are called neural currents, and vascular currents, and they depend for their continuance upon the exercise of the other set of currents referred to above, called respiratory currents. All together they are called "Bichat's tripod of life."

These two sets each consist of a triple series of elements. In the neural currents we have, first, afferent, second, efferent, flowing into a third current in the neural sea of the embryonal mass. The elements of the vascular seas are arterial, capillary, and venous currents in continuous connection, which completes the vascular circuit. The propagation of these currental gyres in the fœtus depends on the respiration of the mother. The *pre*dominant impulses of the respiratory movements of the chest are *sub*dominantly propagated by incitement of nervous currents in the neural, vascular, digestory, and nutrient machinery, and would soon terminate, or come to a state of rest, were they not sustained by the repetition of the respiratory acts. The proof of this is at hand, as witnessed in the increased rapidity of the breathing following and agreeing with increased voluntary movements, mental or bodily. . . . .

In embryology, as in other fields of study, continued and repeated observations tend to correct and supplant the descriptions of former observers, to such extent as to embarrass those who endeavor to follow the authors of any particular period, without being conversant with the whole line of observation and record. These developments have been going on since Aristotle with his "punctum saliens" (now "vascular area"); Fabricus and his "cicatricula" (now "blastoderm"); Harvey's "eye of the egg," and "colliquamentum" (now "blastoderm"); Wolfe, who laid the foundation of modern embryology and modern physiology, with his "vis essentialis"; Haller's "organized concrete"; and Doellinger, who induced Pander to take up the study of the fecundated hen's eggs, who gave us his "blastoderm" and the three "embryonal layers" or "embryonal sheets" called serous, mucous, and vascular, (now modified into "epiblast," "mesoblast, and "hypoblast").

All these taken with Von Baer's statements and scholia, through which he interprets the morphological significance of embryological facts, are of great and lasting importance. Since his time these rectifications have continued through the labors of Remak, Rathke, A. Thompson, and others, till we come to Kolliker and confreres; and they are now undergoing renewed elucidation and annotation at the hands of Foster and Balfour, through whom we look for great advances and rectifications of our conceptions of embryological processes, when they shall have completed and published their work on "The Elements of Embryology," in three parts, the first of which is before the profession already. By all these helps and direct practical observations, we learn that the blastoderm in the egg of the hen divides as follows: into, first, the "opaque area," and the "pellucid" or "transparent area"; second, between these the "vascular area" arises from the mesoblast, which comes from "formative cells" which emerge into sight in the "pellucid area" at the edge of the "hypoblast," which is here separate from the "epiblast," while it at first is in close contact everywhere else. When the accumulation of these "formative cells" becomes considerable, the "mesoblast" splits into the "somatopleure" above, and the "splanchnopleure" below, the first being subsequently differentiated into body-walls, and the second into splanchnic-walls, or visceral walls. From foldings and reduplications and closing of these folds and duplicatures, the tissues and the organs of the whole body arise. The embryo forms in the border land of disputed dominion between the "opaque" and "pellucid" areas, ever emerging from the perfectly transparent "zona pellucida" in the form of a nebulous fleck of milky cloud-mass, as the first sensible indication of its presence and proportions. The "area opaka" is superposed upon the "area pellucida," forming at first a perfect circle, or flattened disk, that steadily enlarges and elongates by excess of increment of growth near opposite edges of this circle, where the future head-fold and tail-fold mark the points of most rapid increase of the blastoderm; changing its rounded disk form to an ovoid, constricted, bulbous cylinder, and then to an irregular, recurved figure, now cognisable as the elongated body of the embryo. The morphological changes of generation and replacement of the deciduous embryonal bodies and processes that constitute the intra-oval development of the machinery of function, are too complicated, manifold, and occult, to admit of consecutive delineation of detail in demonstrable clearness, within the limits of a paper such as this, and the time at command. Suffice it to say, that the next stage of embryonal development consists in the appearance of masses of formative corpuscles, which rapidly accumulate on the inner face of the "somatopleure," and immediately chop up into the "proto-vertebræ"; beginning at the site of the future third or fourth cervical vertebra. This chopping up now proceeds from this point toward both ends of the vertebral column, each division serving as a fore-step in the formation of tissue, by laying out the "proto-vertebræ" successively into "bone-plates," "muscle-plates," and "nerve-plates;" from which, by a series of metamorphoses, the bones, muscles, and nerves are developed and correlated together as the perfected body of the fœtus.

The behavior of the splanchnopleure in the production of the viscera of the fœtus, consists of a less complicated series of steps to effect the production of the blood-making apparatus. Instead of being chopped up into masses of formative corpuscles and into proto-vertebræ which differentiate into bone-plates, muscle-plates, and nerve-plates, as occurs in the somatopleure, the differentiation of the splanchnopleure into digestory, circulatory, and respiratory tracts, consists of a series of foldings or duplications of the circular sheet (splanchnopleure), the closing in of which at the two ends marking the head-fold and the tail-fold of the splanchnopleure, produces the two blind ends or *cul-de-sacs*, called the fore-gut and the after-gut, in simple cylindrical form, reaching toward each other with trumpet-like presentment of caliber, the back edges of whose lips fuse together and join the fore-gut and after-gut in one continuous membrance, while the lower border of the trumpet-like lips of the forward and after *cul-de-sacs* unite, presenting a large hernia-like, or limb-like, bulging, balloon-shaped stalk, connecting the fore gut and after-gut in one continuous, irregular, tri-lobed, sausage-like cylinder. The neck of this last formed pendulous, pear-shaped portion that embraces not only the pellucid area, but the entire yolk, or vitellus, is named the splanchno-stalk. On either side of the fore-gut a process of budding swells out, producing the foundation of the right and left future lung, by which the fore-gut becomes contracted at the territory between these two growing bodies. Below this point of contraction the caliber of the gut enlarges abruptly and considerably, sending out on one side a proliferation of a considerable mass to lay the foundation of the future liver, and at the lower end of this enlargement another mass of formative corpuscles destined to be metamorphosed into the pancreas. The end-gut or after-gut is developed in the direction toward the fore-gut, and by a series of reduplications of its caliber becomes differentiated into the rectum, colon, ilium, and jejunum, to join the duodenum, and thus complete the digestory tract, or "*prima via.*"

An accumulation of formative corpuscles arises in the mesoblast near the site of the hind-gut, called the "*genital ridge.*" From a furrow, duplication, or fold in this mass,

"Muller's duct" arises, that becomes differentiated successively into "Wolfian duct," "Wolfian body," ureter and kidney. Sometimes the "Wolfian duct" opens into the cloaca separately from the ureter, and at others by a common mouth. These ducts are the first solid rods of formative corpuscles, and afterward are perforated by an endotheliated, almost indistinguishable tube; the future excretory "Wolfian duct," or ureter. A further tumulus of formative corpuscles projects from the epithelial surface of the Wolfian bodies, which lays the foundation of the genital apparatus; in the female, called "par-ovarium"; in the male, the "par-epididymus" of Henle, and the "paradidymus" of Waldeyer, the so-called homologa of each other.

Embryologists are still in doubt as to the regularity of the specializations of the embryonal bodies that are worked into the folds and differentiations of folds. This is evidenced by their persisting in disputing as to the mesoblastic and epiblastic elements continuing to act in the somatopleure as distinctly epiblastic or mesoblastic; and also in denying that the splanchnopleure can act as a body which conjoins the mesoblastic and hypoblastic function by proliferating the mesoblastic and hypoblastic elements as such, and not as the product of the compound body definitely named splanchnopleure, from which the parenchyma and the epithelium of the viscera arise. The bastoderm has bi-laminar, tri-laminar, and quadri-laminar aspects at the varying stages of the differentiations into epiblast and hypoblast, between which the mesoblast is introduced, soon to split into the bi-laminar membranes somatopleure and splanchnopleure, which is a quadri-laminar sheet immediately before splitting into these bi-laminar sheets.

With your permission I will now close this paper in the language of another, as taken from an essay entitled "The New Body and the New Birth."

At his first birth MAN is a helpless animal, whose members and faculties are held together, and whose actions are directed by SELF-LOVE, the love whose chief aim is to *get* good. Out of SELF-LOVE gradually grows SOCIAL-LOVE, which aims to *confer* pleasure and to *do* good from mixed motives, one of which is vanity, or the wish to be admired. Next in order of development is DIVINE LOVE; which seeks the good and feels the good of others from a divine joy or sympathy in the welfare of others. This holy love or sympathy gradually brings man into sympathy with God.

In proportion as a man's soul is filled with Divine Love, it lives in the good that it does to others, or that it sees other vital organisms enjoy. True marriage (which implies fidelity to the trusts and obligations that belong to marriage), is the main entrance to Divine Love. Marriage gives to man a body, composed not of will-bound atoms, but of free persons, or of wife and children and children's children. Many a husband feels the breeding-sickness during his wife's pregnancy; and more than one woman has felt the pains of a husband or child when he was dying in a distant place. Divine Love, however, holds but a divided dominion anywhere among men on earth, and men's highest experiences show only its latent tendencies rather than its ripe, vivifying power. Yet it is certain that we are capable of Divine Love; that a common and mutual love causes mutuality of life among its sharers; and that by loving in God's way either persons, or principles and qualities that God loves, we actually live in them, or conjoin them to our spirit's body.

Being infinite in its nature, Divine Love can evermore spread and grow intenser wherever it is kindled. It can enable millions to live each a distinct life in what is substantially the same body, as a whole cathedral full of persons can each lose himself in the strains of a thousand harmonious voices, and appropriate the whole concert. Analogy indicates that Divine Love, after enabling one to live divinely in a smaller body of harmonized souls, can then enable him to incarnate himself in harmonized generations and nations and worlds. Divine Love is ubiquitous, and can give even the human soul a body whose faculties grasp everything worthy of its heart; a body whose birth into new heavens never ceases, whose growth is eternal.—*Transaction American Dental Convention.*

